# Hard and Soft Tissue Asymmetry in Patients with Skeletal Class III Malocclusion: A Cone-Beam Computed Tomography Study

**DOI:** 10.3390/diagnostics13050869

**Published:** 2023-02-24

**Authors:** Tim King Man Tam, Runzhi Guo, Hao Liu, Yifan Lin

**Affiliations:** 1 Division of Pediatric Dentistry and Orthodontics, Faculty of Dentistry, The University of Hong Kong, Hong Kong SAR, China; 2 Department of Orthodontics, Peking University School and Hospital of Stomatology, Beijing 100081, China

**Keywords:** facial asymmetry, skeletal Class III malocclusion, soft tissue thickness, cone-beam computed tomography

## Abstract

This study aims to investigate hard and soft tissue asymmetry in skeletal Class III patients to elucidate how soft tissue thickness alters overall asymmetry and whether menton deviation is correlated with bilateral differences in hard and soft tissue prominence and soft tissue thickness. The cone-beam computed tomography data of 50 skeletal Class III adults were divided based on menton deviation into symmetric (*n* = 25; deviation ≤ 2.0 mm) and asymmetric (*n* = 25; deviation > 2.0 mm) groups. Forty-four corresponding hard and soft tissue points were identified. Bilateral hard and soft tissue prominence and soft tissue thickness were compared using paired t-tests. The correlations between bilateral differences in these variables and menton deviation were examined using Pearson’s correlation analysis. In the symmetric group, no significant bilateral differences in soft and hard tissue prominence and soft tissue thickness were observed. In the asymmetric group, both hard and soft tissue prominence were significantly greater on the deviated side than the non-deviated side at most of the points; however, no significant differences in soft tissue thickness were detected except at point 9 (ST9/ST’9, *p* = 0.011). The difference of hard and soft tissue prominence at point 8 (H8/H’8 and S8/S’8) was positively correlated with menton deviation, whereas the soft tissue thickness at point 5 (ST5/ST’5) and point 9 (ST9/ST’9) was negatively correlated with menton deviation (*p* = 0.05). Soft tissue thickness does not affect overall asymmetry in the presence of underlying hard tissue asymmetry. Soft tissue thickness at the centre of the ramus may be correlated with the degree of menton deviation in patients with asymmetry; however, this correlation needs to be confirmed by further studies.

## 1. Introduction

Facial symmetry is characterised by paired facial structures arranged equidistantly on opposite sides of the mid-sagittal plane (MSP). A more symmetrical face is correlated with a higher degree of attractiveness [1,2,3,4,5,6]. Asymmetry of the face manifests in three dimensions, which can be quantified using different approaches. Mild degrees of facial asymmetry are common in the human population and are accepted as the natural result of normal growth and development [7]. However, moderate to severe facial asymmetry can lead to negative impacts in terms of oral functions [8], facial attractiveness [9], and psychosocial well-being [10].

Different causal factors contribute to facial asymmetry, including genetic factors and acquired factors such as pathology, trauma or environmental disturbances [11,12,13]. Furthermore, facial asymmetry can be ascribed to dental, skeletal, muscular or functional causes [14]. Therefore, both the facial skeleton and the overlying soft tissue may affect the severity of perceivable facial asymmetry. Theoretically, the overall effect can be additive, subtractive or neutral. In other words, soft tissue may aggravate, alleviate or have no effect on asymmetry caused by underlying hard tissue. Kim et al. found that the degree of soft tissue asymmetry was lower than that of bone asymmetry in cases of chin deviation [15]. However, de Lima et al. concluded that soft tissue did not compensate or disguise underlying skeletal asymmetry in the mandibular area [16]. Thus, there is currently no conclusive evidence to support whether soft tissue affects facial asymmetry in the presence of underlying hard tissue asymmetry.

In humans, facial asymmetry is most prevalent in the lower face and is closely related to skeletal Class III malocclusion, with menton deviation being the most common feature [11,17,18]. Because facial asymmetry may stem from both hard and soft tissues, the correction of facial asymmetry via combined orthodontic and orthognathic surgery requires the meticulous pre-operative evaluation of hard and soft tissue components to arrive at a definite diagnosis and to select the appropriate treatment modalities. Therefore, a good understanding of the hard and soft tissue relationship in patients with facial asymmetry is of utmost importance for achieving successful treatment outcomes and improving treatment predictability.

The aim of the current study was to compare bilateral hard and soft tissue prominence in skeletal Class III patients to elucidate how soft tissue thickness may alter overall asymmetry in the presence of underlying hard tissue asymmetry, and to identify potential correlations between menton deviation and these bilateral differences.

## 2. Materials and Methods

This study was approved by the Institutional Review Board of the University of Hong Kong/Hospital Authority Hong Kong West Cluster (Reference number: UW 22-251). Informed consent was obtained from all subjects involved in the study.

### 2.1. Sample Size Calculation

A sample size calculation was performed using G*Power software (version 3.1.9.7, Kiel University, Kiel, Germany). With reference to a previous study, we determined that a total sample size of 20 of subjects would be sufficient to detect 2.0 mm differences between deviated and non-deviated sides (standard deviation = 3.0 mm, alpha = 0.05, power = 0.80) [16].

### 2.2. Patient Inclusion

The inclusion criteria were: (1) at least 18 years old; (2) Class III skeletal pattern (ANB < 0°); and (3) absence of a premature contact leading to a mandibular displacement. Patients with cleft lip and palate, craniofacial syndromes, a history of facial soft tissue derangement or a history of craniofacial surgery were excluded. Based on these criteria, 50 cone-beam computed tomography (CBCT) scans were included in this study. These patients were indicated for combined orthodontics and orthognathic surgery treatment due to having a skeletal Class III dentofacial deformity. Pre-operative CBCT scans were taken to provide a three-dimensional (3D) view of the entire craniofacial anatomy and help with diagnosis and treatment planning.

### 2.3. Data Acquisition and Assessment

A CBCT device (Planmeca ProMax^®^ 3D Mid, Planmeca Oy, Helsinki, Finland) was used to capture 3D scans of the facial skeleton with a resolution of 400 µm, and a field of view of 20.1 × 17.4 cm, 120 kV, 8.0 mA and 1155 mGy × cm^2^. During the scanning procedure, the patients were standing straight with a natural head position and were instructed to bite in the intercuspal position with the lips in a repose position.

**Figure 1:**
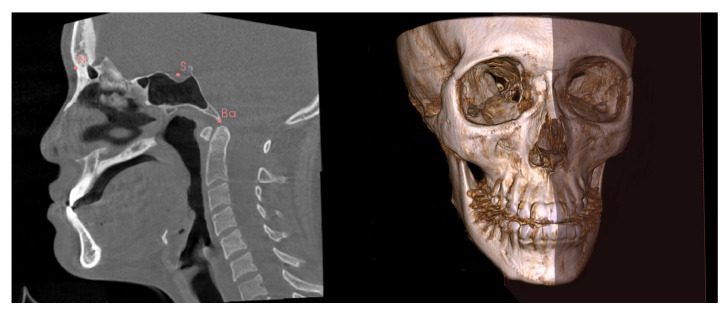
Identification of the N (Nasion), S (Sella), and Ba (Basion) for construction of the mid-sagittal plane (MSP). The MSP bisects the face into left and right halves.

The CBCT scans in the Digital Imaging and Communications in Medicine format were then imported into 3D Slicer software [19]. Using the multiplanar view, three anatomic landmarks (the Nasion, Sella and Basion) were used to construct an MSP for each scan (Figure 1). The MSP was used as a reference plane to define the true plane, which was perpendicular to the horizontal plane, and to bisect the face into left and right halves (Figure 1). The menton point was defined to assess menton deviation.

Patients with the perpendicular distance from the menton point to the MSP ≤ 2.0 mm or >2.0 mm were assigned to the symmetric and asymmetric groups, respectively. Fifty patients were divided into two groups: the symmetric group (*n* = 25) and the asymmetric group (*n* = 25). The side of menton deviation was referred to as the deviated side, and the other side was referred to as the non-deviated side.

**Figure 2:**
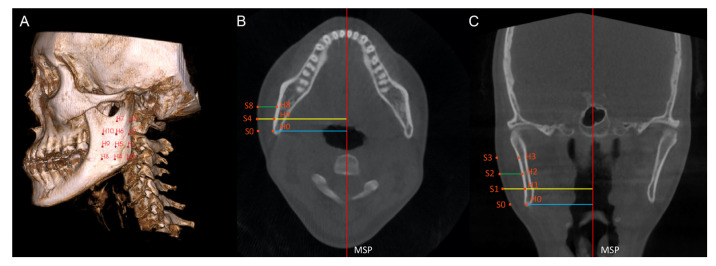
(**A**) Hard tissue points H0 to H10 are shown on the ramus. (**B**,**C**) Hard tissue points and the corresponding soft tissue points are identified on the axial (**B**) and coronal (**C**) slices. The axial and coronal slices show the measurement of hard tissue prominence (blue line), soft tissue prominence (yellow line), and soft tissue thickness (green line). MSP, mid-sagittal plane.

On each scan, 44 anatomic hard tissue (H0 to H10 and H’0 to H’10) and soft tissue points (S0 to S’10 and S’0 to S’10) were identified as follows: on the multiplanar view, the first point located was the hard tissue Gonion (H0) on the deviated side. With reference to H0, 10 additional hard tissue points (H1 to H10) were identified on the bilateral mandibular ramus and body (Table 1), encompassing a large portion of the ramus area (Figure 2A). On the coronal and axial slices, soft tissue points (S0 to S10) were identified by extending each hard tissue point (H0 to H10) perpendicularly from the MSP to the outermost soft tissue contour of the face (Figure 2B,C). Hence, 11 pairs of corresponding soft tissue and hard tissue points were created. Similarly, 11 pairs of points (H’0 to H’10; S’0 to S’10) were created on the non-deviated side of the scan.

The hard tissue prominence was defined as the perpendicular distance from a hard tissue point to the MSP; the soft tissue prominence was defined as the perpendicular distance from a soft tissue point to the MSP. The soft tissue thickness (ST0 to ST10; ST’0 to ST’10) was calculated by subtracting the hard tissue prominence from the corresponding soft tissue prominence. The demonstrations of the points and the measurements are shown in Table 1.

### 2.4. Statistical Analysis

To assess the intra-examiner reliability, 10 CBCT scans were randomly selected from each group to be remeasured 2 weeks later. The Shapiro–Wilk test was used to assess the normality of the data. For intra-group comparisons, paired *t*-tests were used to detect bilateral differences in hard and soft tissue prominence and soft tissue thickness within the asymmetric and symmetric groups. Pearson’s correlation analysis was used to assess the relationships between the absolute amount of menton deviation and bilateral differences in hard and soft tissue prominence and soft tissue thickness, respectively, in both of the groups. Statistical analyses were conducted using SPSS software (version 27.0; IBM, Armonk, NY, USA). A significance level of 0.05 was adopted.

## 3. Results

The sample consisted of 50 CBCT scans of skeletal Class III individuals. Demographic information of the subjects is presented in Table 2. Of the 50 subjects, 26 were male (52.0%), and 24 were female (48.0%), and the patients’ ages ranged from 18.2 to 39.6 years, with a mean of 24.42 years. Twenty-five patients had menton deviations > 2.0 mm and were classified as asymmetric (50.0%); 25 patients had menton deviations ≤ 2.0 mm and were classified as symmetric (50.0%). The menton deviation direction was towards the left side in 28 (56.0%) patients and towards the right side in 22 (44.0%) patients.

The intraclass correlation coefficient showed a high level of agreement for all parameters (range 0.77–0.98). Intra-group comparisons within symmetric and asymmetric groups are presented in Table 3. The distances from 11 hard tissue points and 11 corresponding soft tissue points to the MSP and the soft tissue thickness were compared between the deviated and non-deviated sides. For the symmetric group, bilateral comparisons revealed no significant differences in hard tissue prominence, soft tissue prominence, and soft tissue thicknesses (*p* > 0.05). For the asymmetric group, the distances from seven hard tissue points (H0, H4, H5, H6, H8, H9, and H10) and eight soft tissue points (S0, S1, S4, S5, S6, S8, S9, and S10) on the deviated side to the MSP were significantly greater than the corresponding distances on the non-deviated sides (*p* < 0.05). Among these points, point 8 showed the greatest bilateral hard (4.26 mm, *p* < 0.001) and soft tissue differences (4.29 mm, *p* < 0.001). Only the soft tissue thickness at point 9 (ST9/ST’9) was statistically greater at the non-deviated side than at the deviated side (*p* = 0.011); however, the difference (0.96 mm) may not be clinically significant. Regarding the correlation analysis, the differences of bilateral hard tissue and soft tissue prominence at point 8 (H8/H’8 and S8/S’8) were positively correlated with the menton deviation, whereas the differences of soft tissue thickness at point 5 (ST5/ST’5) and point 9 (ST9/ST’9) were negatively correlated with menton deviation in the asymmetric group (Table 4).

## 4. Discussion

The aims of this study were to compare the bilateral prominence of hard and soft tissues in skeletal Class III patients with and without asymmetry and to determine whether the menton deviation is correlated with bilateral differences in hard and soft tissue prominence and soft tissue thickness. The unequal bilateral prominence of hard tissue in the transverse dimension indicates skeletal asymmetry, while that of soft tissue reflects the degree of facial asymmetry detected by the naked eye. Soft tissue thickness can be a critical factor influencing the visual perception of facial symmetry due to its potential capacity to camouflage or exacerbate the visual effect of skeletal asymmetry.

The question of whether such capacity exists has been discussed in several studies; however, conflicting conclusions have been reported. de Lima et al. found that only one point in the mandible reflected a statistically significant difference in bilateral soft tissue thickness, and concluded that soft tissue thickness does not compensate for underlying hard tissue asymmetry [16]. Other studies have shown that facial soft tissue disguises skeletal hard tissue asymmetry, despite using different methodologies, such as different methods to quantify asymmetry and investigating different parts of the face [15,20,21].

In the current study, the target population was skeletal Class III patients, because the Class III skeletal pattern is associated with a higher prevalence of asymmetries than other skeletal patterns, with asymmetries in the mandibular region being most common. Mandible structures, such as the gonial angle, mandibular ramus and chin, frequently present with asymmetry [17,22,23]. Therefore, these structures hold great potential as targets for evaluating and quantifying asymmetry.

In the current study, the menton deviation from the MSP was used as a parameter to separate subjects into an asymmetric group (>2 mm) and a symmetric group (≤2 mm), as previously suggested [24]. The side towards which the menton deviation occurred was designated as the deviated side, and the opposite side was designated as the non-deviated side. The results showed that the asymmetric group had more prominent hard tissue contours on the deviated side than on the non-deviated side for all 11 points, with the prominence at seven points (H0, H4, H5, H6, H8, H9, and H10) showing statistically significant differences. These points were more inferiorly and anteriorly located than those that did not show a statistically significant difference, with point 8 exhibiting the greatest bilateral discrepancy. Thus, a gradient of increasing asymmetry was observed in the direction from the superior to the inferior and the posterior to the anterior of the face, consistent with several previous studies [16,17,20,25,26,27]. Regarding the soft tissue prominence, the asymmetric group had significant bilateral differences at soft tissue points corresponding to hard tissue points with significant differences. That is, the hard tissue points that displayed significant asymmetry were usually accompanied by significantly asymmetric soft tissue prominence. The symmetric group did not show any statistically significant results in this regard, as expected.

Furthermore, no significant correlations between bilateral differences in hard and soft tissue prominence and menton deviation were observed in the symmetric group. In the asymmetric group, the differences of hard tissue and soft tissue prominence at point 8 were positively correlated with menton deviation, which is consistent with the greatest bilateral difference at point 8. In addition, bilateral soft tissue thickness differences at point 5 and point 9 were negatively correlated with menton deviation in the asymmetric group. Notably, point 5 and point 9 lie in the centre of the ramus, covered by the masseter muscle. In the asymmetric group, variation in the shape of the rami as well as the morphology of the masseter muscles may lead to appreciable asymmetry in soft tissue thickness. Moreover, patients with significant asymmetry frequently exhibit a unilateral crossbite, which negatively affects masticatory muscle performance during mastication, resulting in poor muscular coordination and potentially contributing to soft tissue thickness asymmetry [28,29]. The statistically significant correlation indicates that patients with a greater degree of chin deviation may present with more significant bilateral differences in soft tissue thickness; however, considering the lack of significance in the majority of measurements of soft tissue thickness, the degree and extent of the resulting asymmetry should be verified by further studies.

Whether soft tissue will camouflage or exacerbate underlying skeletal asymmetry is a critical question. When treating orthognathic patients presenting with facial asymmetry, soft tissue asymmetry may remain after surgically correcting the skeletal structures to improve hard tissue asymmetry, leading to compromised outcomes and patient dissatisfaction [30,31,32,33,34,35]. Lee et al. investigated the influence of mandibular surgery on asymmetric mandibular prognathism patients and reported that while skeletal asymmetry was the major contributing factor, soft tissue thickness still played a role in creating overall asymmetry [20]. Therefore, investigations of soft tissue thickness in patients with asymmetry are warranted. Furthermore, Lee et al. reported that soft tissue was thinner on the deviated side of the mandible, compensating for hard tissue asymmetry [20]. Thus, the degree of craniofacial skeleton asymmetry may be more severe than that estimated from patients’ photographs [20]. Similarly, Kim et al. reported that soft tissue tends to compensate for hard tissue asymmetry in the ramal and corpus regions, and in asymmetric subjects there was usually less asymmetry in soft tissue than in hard tissue, except for lip cant and lip cheilion height differences [15]. In contrast, Hwang et al. found larger bilateral differences in soft tissue than in hard tissue, even in normal occlusion subjects, implying that soft tissue alters the overall symmetry of the face, regardless of the direction of the alteration, and its effect should not be underestimated [36].

The current study primarily investigated the ramal area because it contributes to the bilateral contour outlining the face, which affects the perception of facial asymmetry in daily life. The results of the current study oppose the view that soft tissue may compensate for underlying skeletal asymmetry but support the findings of de Lima et al. [16]. It showed that despite the hard and soft tissue asymmetry between the deviated and non-deviated sides in the asymmetric group, there is insufficient evidence to suggest whether soft tissue camouflages or aggravates whole facial asymmetry.

From a different perspective, soft tissue behaviours on asymmetric skeletal structures might differ depending on their location in the face. Kim et al. found that hard tissue asymmetry in the form of chin deviation, frontal ramal inclination difference, and frontal corpus inclination difference were accompanied by a smaller degree of respective soft tissue asymmetry, while soft tissue parameters, such as lip cant and lip cheilion height differences showed greater asymmetry than their underlying hard tissue [15]. The soft component of the face encompasses numerous craniofacial muscles of varying sizes, activities and insertion points. Therefore, soft tissue behaviour may be complicated by factors beyond underlying skeletal asymmetries, such as asymmetric muscular activities and parafunctional habits. The current understanding of how soft tissue and hard tissue interact with each other in the presence of asymmetry is limited; further evidence is needed to advance our understanding of this topic.

One limitation of the study is that the soft tissue thickness of the mandibular ramus was defined and examined in relation to the MSP in this study. It is important to note that the ramus is seated at an angle to the MSP such that the inter-distance between the outer contour of the left and right rami decreases from posterior to anterior. Alternatively, the soft tissue thickness of a particular hard tissue point on the ramus can be defined as the shortest distance from that point to the soft tissue ‘plane’, also called the signed distance function. Different investigation approaches may contribute to the contradictory findings of various studies. In addition, the selected landmarks do not provide a comprehensive representation of the entire facial region. A volume-based superimposition approach is necessary to gain a complete understanding of the asymmetry of soft tissue thickness.

## 5. Conclusions

In the current study, skeletal Class III asymmetric patients showed greater hard and soft tissue prominence on the side of menton deviation. However, the absence of significant differences in soft tissue thickness suggests that soft tissue may not alter the overall asymmetry in the presence of underlying skeletal asymmetry. Soft tissue thickness at the centre of the ramus may be correlated with the degree of menton deviation in patients with asymmetry; however, this correlation needs to be confirmed by further studies.

## Figures and Tables

**Table 1:** Descriptions of the points and the measurements used in this study.

	Description
Points	
H0	Point corresponding to the Gonion point on the deviated side.
H1	Point located on the external cortical bone of the mandible, on the same coronal slice as H0, and 10 mm above H0 on the axial slice.
H2	Point located on the external cortical bone of the mandible, on the same coronal slice as H0, and 10 mm above H1 on the axial slice.
H3	Point located on the external cortical bone of the mandible, on the same coronal slice as H0, and 10 mm above H2 on the axial slice.
H4	Point located on the external cortical bone of the mandible, on the same axial slice as H0, and 10 mm anterior to H0 on the coronal slice.
H5	Point located on the external cortical bone of the mandible, on the same coronal slice as H4, and 10 mm above H4 on the axial slice.
H6	Point located on the external cortical bone of the mandible, on the same coronal slice as H4, and 10 mm above H5 on the axial slice.
H7	Point located on the external cortical bone of the mandible, on the same coronal slice as H4, and 10 mm above H6 on the axial slice.
H8	Point located on the external cortical bone of the mandible, on the same axial slice as H4, and 10 mm anterior to H4 on the coronal slice.
H9	Point located on the external cortical bone of the mandible, on the same coronal slice as H8, and 10 mm above H8 on the axial slice.
H10	Point located on the external cortical bone of the mandible, on the same coronal slice as H8, and 10 mm above H9 on the axial slice.
S0–S10	Extension of hard tissue point (H0 to H10) perpendicularly from the MSP to the outermost soft tissue contour of the face.
H’0–H’10	Hard tissue points on the non-deviated side of the mandible, corresponding to H0 to H10.
S’0–S’10	Soft tissue points on the non-deviated side, corresponding to S0 to S10.
Measurements	
Hard tissue prominence (mm)	The perpendicular distance from a hard tissue point to the MSP.
Soft tissue prominence (mm)	The perpendicular distance from a soft tissue point to the MSP.
Soft tissue thickness (mm)	The distance between a hard tissue point and a soft tissue point perpendicular to the MSP.
Menton deviation (mm)	The perpendicular distance from the menton point to the mid-sagittal plane.

**Table 2:** Demographic characteristics of included subjects.

	Symmetric Group	Asymmetric Group
Variables		
Patient (*n*)	25	25
Gender (Male/Female)	12 M, 13 F	14 M, 11 F
Age (year), mean ± SD	23.65 ± 5.12	25.18 ± 4.77
menton deviation (mm), mean ± SD	0.87 ± 0.67	4.26 ± 1.69
Side of menton deviation (*n*)	13 L, 12 R	15 L, 10 R

L, left-sided; R, right-sided.

**Table 3:** Comparison between deviated and non-deviated sides in the symmetric and asymmetric groups.

	Symmetric Group (*n* = 25)						Asymmetric Group (*n* = 25)					
Variables	Deviated Side	SD	Non-Deviated Side	SD	Mean Difference	*p* Value #	Deviated Side	SD	Non-Deviated Side	SD	Mean Difference	*p* Value #
Hard tissue prominence (mm)												
H0/H’0	48.94	4.57	48.95	2.54	−0.02	0.985	52.08	3.15	48.51	3.89	3.57	0.002 *
H1/H’1	48.68	3.63	49.31	2.42	−0.63	0.492	51.21	3.22	48.94	3.58	2.27	0.054
H2/H’2	50.08	3.53	50.52	2.96	−0.43	0.670	52.29	3.01	50.76	3.46	1.53	0.175
H3/H’3	51.65	3.64	52.53	3.27	−0.88	0.388	53.31	2.85	52.25	2.89	1.06	0.311
H4/H’4	47.06	3.45	46.87	2.39	0.19	0.843	50.40	2.93	46.70	3.20	3.70	0.002 *
H5/H’5	47.88	3.42	47.74	2.82	0.14	0.880	50.78	2.89	47.30	3.17	3.49	0.002 *
H6/H’6	48.96	3.56	48.71	3.50	0.25	0.798	51.39	3.10	48.99	2.91	2.40	0.027 *
H7/H’7	49.53	4.11	49.47	4.03	0.07	0.947	51.42	2.98	49.80	2.41	1.62	0.097
H8/H’8	41.56	10.87	44.50	2.21	−2.94	0.199	47.96	3.36	43.70	2.54	4.26	<0.001 *
H9/H’9	44.77	3.52	44.60	2.83	0.17	0.849	47.76	3.24	44.07	2.78	3.69	0.001 *
H10/H’10	45.55	3.58	45.29	3.38	0.26	0.777	47.52	3.54	44.85	2.60	2.67	0.011 *
Soft tissue prominence (mm)												
S0/S’0	60.35	4.26	59.81	4.10	0.54	0.615	61.50	3.25	58.33	3.71	3.17	0.006 *
S1/S’1	64.12	3.80	63.88	4.04	0.23	0.781	65.26	3.16	62.79	3.71	2.46	0.028 *
S2/S’2	67.20	3.46	66.85	4.37	0.35	0.700	68.44	3.62	66.37	3.62	2.07	0.062
S3/S’3	69.25	2.88	69.21	4.49	0.04	0.965	70.09	3.80	68.86	3.85	1.22	0.273
S4/S’4	62.15	3.46	61.72	3.92	0.43	0.625	63.68	3.35	60.13	3.06	3.56	0.001 *
S5/S’5	65.94	3.15	65.23	4.02	0.70	0.387	66.89	3.17	64.14	3.05	2.75	0.004 *
S6/S’6	68.22	3.56	67.79	4.14	0.44	0.555	69.07	3.44	67.05	3.23	2.02	0.039 *
S7/S’7	69.67	3.42	69.82	4.71	−0.15	0.853	70.16	3.33	69.17	3.31	0.99	0.297
S8/S’8	62.38	3.67	61.62	4.22	0.76	0.397	63.66	4.13	59.37	2.94	4.29	<0.001 *
S9/S’9	65.20	3.25	64.91	4.28	0.29	0.711	66.33	3.73	63.60	3.27	2.73	0.005 *
S10/S’10	67.85	3.03	67.40	4.56	0.44	0.555	68.32	3.65	66.32	3.42	2.00	0.045 *
Soft tissue thickness (mm)												
ST0/ST’0	11.41	2.96	10.86	3.36	0.55	0.114	9.42	1.99	9.82	2.89	−0.40	0.279
ST1/ST’1	15.44	3.00	14.57	2.77	0.87	0.077	14.05	1.92	13.86	2.79	0.19	0.577
ST2/ST’2	17.12	2.45	16.34	2.59	0.78	0.131	16.15	2.12	15.61	2.69	0.54	0.052
ST3/ST’3	17.60	2.39	16.68	2.32	0.92	0.058	16.78	2.39	16.61	3.23	0.17	0.647
ST4/ST’4	15.10	2.46	14.85	2.35	0.25	0.301	13.29	1.88	13.43	2.77	−0.15	0.725
ST5/ST’5	18.06	2.26	17.50	1.84	0.56	0.124	16.11	2.21	16.84	2.99	−0.74	0.116
ST6/ST’6	19.26	2.26	19.08	1.68	0.19	0.711	17.68	2.48	18.06	3.11	−0.38	0.364
ST7/ST’7	20.14	2.31	20.36	1.86	−0.22	0.619	18.74	2.75	19.37	3.10	−0.62	0.162
ST8/ST’8	20.82	11.84	17.12	2.64	3.70	0.123	15.70	2.03	15.67	2.64	0.03	0.919
ST9/ST’9	20.43	2.87	20.32	2.07	0.12	0.735	18.56	2.38	19.52	2.98	−0.96	0.011 *
ST10/ST’10	22.29	2.48	22.11	1.96	0.18	0.547	20.80	2.73	21.47	3.08	−0.67	0.159

# Paired Student’s *t*-Test. * *p* < 0.05.

**Table 4:** Correlation between menton deviation and bilateral differences in the symmetric and asymmetric groups.

	Symmetric Group (*n* = 25)		Asymmetric Group (*n* = 25)	
Variables	Correlation Coefficient	*p* Value	Correlation Coefficient	*p* Value
Hard tissue difference				
H0/H’0	0.166	0.427	−0.027	0.897
H1/H’1	0.135	0.519	−0.043	0.838
H2/H’2	0.146	0.485	−0.157	0.452
H3/H’3	0.023	0.915	−0.180	0.388
H4/H’4	0.224	0.281	0.187	0.371
H5/H’5	0.183	0.382	0.215	0.302
H6/H’6	0.094	0.655	0.047	0.824
H7/H’7	0.125	0.551	−0.007	0.972
H8/H’8	0.356	0.062	0.451	0.024 *
H9/H’9	0.184	0.379	0.271	0.190
H10/H’10	0.221	0.288	0.173	0.409
Soft tissue difference				
S0/S’0	0.250	0.228	0.152	0.468
S1/S’1	0.299	0.147	−0.017	0.937
S2/S’2	0.204	0.329	−0.122	0.562
S3/S’3	0.187	0.371	−0.142	0.500
S4/S’4	0.245	0.238	0.323	0.116
S5/S’5	0.227	0.276	0.038	0.856
S6/S’6	0.182	0.383	−0.081	0.701
S7/S’7	0.185	0.375	−0.195	0.351
S8/S’8	0.269	0.194	0.412	0.041 *
S9/S’9	0.348	0.088	0.064	0.759
S10/S’10	0.266	0.199	−0.078	0.710
Soft tissue thickness difference				
ST0/ST’0	0.342	0.095	0.252	0.108
ST1/ST’1	0.377	0.063	0.092	0.661
ST2/ST’2	0.067	0.749	0.182	0.384
ST3/ST’3	0.305	0.139	0.084	0.690
ST4/ST’4	0.028	0.895	0.244	0.240
ST5/ST’5	0.026	0.904	−0.416	0.038 *
ST6/ST’6	0.085	0.685	−0.298	0.148
ST7/ST’7	0.048	0.821	−0.303	0.146
ST8/ST’8	−0.337	0.100	−0.252	0.225
ST9/ST’9	0.303	0.141	−0.575	0.003 *
ST10/ST’10	−0.023	0.912	−0.326	0.117

* *p* < 0.05.

## Data Availability

The data presented in this study are available on request from the corresponding author.

## References

[1] Rhodes G., Proffitt F., Grady J.M., Sumich A. (1998). Facial symmetry and the perception of beauty. Psychon. Bull. Rev..

[2] Little A.C., Jones B.C., DeBruine L.M. (2011). Facial attractiveness: Evolutionary based research. Philos. Trans. R Soc. Lond. B Biol. Sci..

[3] Grammer K., Thornhill R. (1994). Human (Homo sapiens) facial attractiveness and sexual selection: The role of symmetry and averageness. J. Comp. Psychol..

[4] Scheib J.E., Gangestad S.W., Thornhill R. (1999). Facial attractiveness, symmetry and cues of good genes. Proc. Biol. Sci..

[5] Penton-Voak I.S., Jones B.C., Little A.C., Baker S., Tiddeman B., Burt D.M., Perrett D.I. (2001). Symmetry, sexual dimorphism in facial proportions and male facial attractiveness. Proc. Biol. Sci..

[6] Jones B.C., Little A.C., Penton-Voak I.S., Tiddeman B.P., Burt D.M., Perrett D.I. (2001). Facial symmetry and judgements of apparent health: Support for a “good genes” explanation of the attractiveness–symmetry relationship. Evol. Hum. Behav..

[7] Claes P., Walters M., Vandermeulen D., Clement J.G. (2011). Spatially-dense 3D facial asymmetry assessment in both typical and disordered growth. J. Anat..

[8] Rossi M., Ribeiro E., Smith R. (2003). Craniofacial asymmetry in development: An anatomical study. Angle Orthod..

[9] Shaw W.C., Rees G., Dawe M., Charles C.R. (1985). The influence of dentofacial appearance on the social attractiveness of young adults. Am. J. Orthod.

[10] Shackelford T.K., Larsen R.J. (1997). Facial asymmetry as an indicator of psychological, emotional, and physiological distress. J. Pers. Soc. Psychol..

[11] Haraguchi S., Iguchi Y., Takada K. (2008). Asymmetry of the face in orthodontic patients. Angle Orthod..

[12] Lundström A. (1961). Some asymmetries of the dental arches, jaws, and skull, and their etiological significance. Am. J. Orthod..

[13] Cheong Y.W., Lo L.J. (2011). Facial asymmetry: Etiology, evaluation, and management. Chang. Gung Med. J..

[14] Bishara S.E., Burkey P.S., Kharouf J.G. (1994). Dental and facial asymmetries: A review. Angle Orthod..

[15] Wang-Sik K., Ki-Heon L., Hyeon-Shik H. (2005). Comparison of asymmetric degree between maxillofacial hard and soft tissue in facial asymmetric subjects using three-dimensional computed tomography. Korean J. Orthod..

[16] Siqueira de Lima L., Brunetto D.P., da Cunha Gonçalves Nojima M. (2019). Evaluation of facial soft tissue thickness in symmetric and asymmetric subjects with the use of cone-beam computed tomography. Am. J. Orthod. Dentofac. Orthop..

[17] Severt T.R., Proffit W.R. (1997). The prevalence of facial asymmetry in the dentofacial deformities population at the University of North Carolina. Int. J. Adult Orthodon Orthognath. Surg..

[18] Good S., Edler R., Wertheim D., Greenhill D. (2006). A computerized photographic assessment of the relationship between skeletal discrepancy and mandibular outline asymmetry. Eur. J. Orthod..

[19] Fedorov A., Beichel R., Kalpathy-Cramer J., Finet J., Fillion-Robin J.C., Pujol S., Bauer C., Jennings D., Fennessy F., Sonka M. (2012). 3D Slicer as an image computing platform for the Quantitative Imaging Network. Magn Reson. Imaging.

[20] Lee S.T., Mori Y., Minami K., An C.H., Park J.W., Kwon T.G. (2013). Does skeletal surgery for asymmetric mandibular prognathism influence the soft tissue contour and thickness?. J. Oral. Maxillofac. Surg..

[21] Huang L., Li Z., Yan J., Chen L., Piao Z.G. (2021). Evaluation of facial soft tissue thickness in asymmetric mandibular deformities after orthognathic surgery. Maxillofac. Plast Reconstr. Surg..

[22] Maeda M., Katsumata A., Ariji Y., Muramatsu A., Yoshida K., Goto S., Kurita K., Ariji E. (2006). 3D-CT evaluation of facial asymmetry in patients with maxillofacial deformities. Oral. Surg. Oral. Med. Oral. Pathol. Oral. Radiol. Endod.

[23] Ajmera D.H., Hsung R.T., Singh P., Wong N.S.M., Yeung A.W.K., Lam W.Y.H., Khambay B.S., Leung Y.Y., Gu M. (2022). Three-dimensional assessment of facial asymmetry in Class III subjects. Part 1: A retrospective study evaluating postsurgical outcomes. Clin. Oral. Investig..

[24] Kim S.J., Lee K.J., Lee S.H., Baik H.S. (2013). Morphologic relationship between the cranial base and the mandible in patients with facial asymmetry and mandibular prognathism. Am. J. Orthod. Dentofac. Orthop..

[25] Kim B.R., Oh K.M., Cevidanes L.H., Park J.E., Sim H.S., Seo S.K., Reyes M., Kim Y.J., Park Y.H. (2013). Analysis of 3D soft tissue changes after 1- and 2-jaw orthognathic surgery in mandibular prognathism patients. J. Oral. Maxillofac. Surg..

[26] Ferrario V.F., Sforza C., Ciusa V., Dellavia C., Tartaglia G.M. (2001). The effect of sex and age on facial asymmetry in healthy subjects: A cross-sectional study from adolescence to mid-adulthood. J. Oral. Maxillofac. Surg..

[27] Nur R.B., Çakan D.G., Arun T. (2016). Evaluation of facial hard and soft tissue asymmetry using cone-beam computed tomography. Am. J. Orthod. Dentofac. Orthop..

[28] Andrade Ada S., Gavião M.B., Gameiro G.H., De Rossi M. (2010). Characteristics of masticatory muscles in children with unilateral posterior crossbite. Braz Oral. Res..

[29] Iodice G., Danzi G., Cimino R., Paduano S., Michelotti A. (2016). Association between posterior crossbite, skeletal, and muscle asymmetry: A systematic review. Eur. J. Orthod..

[30] Li Y., Hu Z., Ye B., Liu Y., Ren X., Zhu S. (2016). Combined Use of Facial Osteoplasty and Orthognathic Surgery for Treatment of Dentofacial Deformities. J. Oral. Maxillofac. Surg..

[31] Vittert L., Katina S., Ayoub A., Khambay B., Bowman A.W. (2018). Assessing the outcome of orthognathic surgery by three-dimensional soft tissue analysis. Int. J. Oral. Maxillofac. Surg..

[32] Wermker K., Kleinheinz J., Jung S., Dirksen D. (2014). Soft tissue response and facial symmetry after orthognathic surgery. J. Craniomaxillofac. Surg..

[33] Ho C.T., Lin H.H., Liou E.J., Lo L.J. (2017). Three-dimensional surgical simulation improves the planning for correction of facial prognathism and asymmetry: A qualitative and quantitative study. Sci. Rep..

[34] Denadai R., Pai B.C., Lo L.J. (2020). Balancing the dental occlusion and facial aesthetic features in cleft orthognathic surgery: Patient-centered concept for computer-aided planning. Biomed. J..

[35] Liao Y.F., Chen Y.F., Yao C.F., Chen Y.A., Chen Y.R. (2019). Long-term outcomes of bimaxillary surgery for treatment of asymmetric skeletal class III deformity using surgery-first approach. Clin. Oral. Investig..

[36] Hwang H.S., Yuan D., Jeong K.H., Uhm G.S., Cho J.H., Yoon S.J. (2012). Three-dimensional soft tissue analysis for the evaluation of facial asymmetry in normal occlusion individuals. Korean J. Orthod..

